# Japanese translation of the Functional Assessment of Cancer Therapy-Breast + 4 (FACT-B + 4) following international guidelines: a verification of linguistic validity

**DOI:** 10.1007/s12282-025-01701-x

**Published:** 2025-05-08

**Authors:** Takahiro Tsukioki, Nozomu Takata, Saya R. Dennis, Kaori Terata, Yasuaki Sagara, Takehiko Sakai, Shin Takayama, Dai Kitagawa, Yuichiro Kikawa, Yuko Takahashi, Tsuguo Iwatani, Fumikata Hara, Tomomi Fujisawa, Tadahiko Shien

**Affiliations:** 1 Department of Breast and Endocrine Surgery, Okayama University Hospital, 2-5-1 Shikata-cho, Kita-ku, Okayama city, Japan; 2 Simpson Querrey Biomedical Research Center, Northwestern University, Evanston, USA; 3 Department of Preventive Medicine Feinberg School of Medicine, Northwestern University, Chicago, USA; 4 Department of Breast and Endocrine Surgery, Akita University Hospital, Akita city, Japan; 5 Department of Breast Surgical Oncology, Social Medical Corporation Hakuaikai Sagara Hospital, Kagoshima city, Japan; 6 Department of Surgical Oncology, Breast Oncology Center, Cancer Institute Hospital of JFCR, Koto City, Japan; 7 Department of Breast Surgery, National Cancer Center Hospital, Chuo City, Japan; 8 Department of Breast Surgical Oncology, National Center for Global Health and Medicine, Shinjuku City, Japan; 9 Department of Breast Surgery, Kansai Medical University Hospital, Osaka city, Japan; 10 Department of Breast Oncology, Aichi Cancer Center Hospital, Nagoya city, Japan; 11 Department of Breast Cancer, Gunma Prefectural Cancer Center, Ota-shi, Japan

**Keywords:** Breast cancer, FACT-B, FACT-B+4, QOL

## Abstract

**Background:**

For breast cancer patients, postoperative lymphedema and upper limb movement disorders are serious complications that absolutely reduce their quality of life (QOL). To evaluate this serious complication, we used “Quick Dash” or “FACT-B”, which can assess a patient's physical, social, emotional, and functional health status. To evaluate their breast cancer surgery-related dysfunction correctly, “FACT-B + 4” was created by adding four questions about “arm swelling'' and “tenderness”. We have translated it into Japanese according to international translation guidelines.

**Methods:**

At the beginning, we contacted FACT headquarters that we would like to create a Japanese version of FACT-B + 4. They formed the FACIT Trans Team (FACIT) following international translation procedures, and then, we began translating according to them. The steps are 1: perform “Forward and Reverse translations” to create a “Preliminary Japanese version”, 2: request the cooperation of 5 breast cancer patients and “conduct a pilot study” and “questionnaire survey”, and 3: amendments and final approval based on pilot study results and clinical perspectives.

**Result:**

In Step1, FACIT requested faithful translation of the words, verbs, and nouns from the original text. In Step2, patients reported that they felt uncomfortable with the Japanese version words such as “numb'' and “stiffness'' and felt that it might be difficult to describe their symptoms accurately. In Step3, we readjusted the translation to be more concise and closer to common Japanese language, and performed “Step1” again to ensure that the translation definitely retained the meaning of the original.

**Conclusion:**

A Japanese version of FACT has existed until now, but there was no Japanese version of FACT-B + 4, which adds four additional items to evaluate swelling and pain in the upper limbs. This time, we have created a Japanese version that has been approved by FACT.

## Background

Breast cancer is the most common malignant tumor in women, and according to the 2019 national cancer registry data in Japan, there were approximately 95,000 breast cancer patients that year, and the number of patients is increasing year by year. Breast cancer treatment is multidisciplinary and combines surgical therapy, drug therapy, and radiation therapy. As prognosis is improving due to developments in imaging technology and drug therapy, future challenges will be to improve cancer survivorship, such as improving post-treatment quality of life (QOL) and help patients return to society.

Some of the major treatment complications that trouble breast cancer survivors are surgery-related complications, such as lymphedema and postoperative pain. In particular, cases involving axillary dissection are accompanied by many side effects, such as pain due to causalgia, lymphedema caused by intercostobrachial nerve damage, limited shoulder movement, and numbness. These complications not only cause pain and functional impairment, but also cause mental stress and significantly impair daily living activities [1–6]. It has been reported that lymphedema occurs in 5.6% of patients who undergo sentinel lymph-node biopsy, and in 20% of patients who undergo axillary dissection, and a meta-analysis has also shown that patients who undergo dissection are approximately four times more likely to develop lymphedema [7, 8]. Treatment for lymphedema includes compression therapy with elastic garments and bandages, manual drainage, and lymphatic anastomosis, but none of these methods is very effective, and many patients suffer from lymphedema for a long time after surgery. As far as we know, there are no prospective studies comparing QOL considering lymphedema in patients who underwent sentinel node biopsy and axillary dissection, yet axillary dissection clearly increases the rate of lymphedema and thus decreases QOL.

In recent years, clinical studies have examined new surgical techniques for prevention of lymphedema, such as reducing the extent of axillary dissection [9, 10], sentinel node biopsy-guided axillary dissection in patients with negative lymph nodes after preoperative chemotherapy [11, 12], and tailored axillary surgery [13–18]. However, the long-term outcome of lymphedema incidence, and the extent to which these procedures improve the patient’s QOL, are unclear. In addition, there are still cases that require lymphatic dissection, and prevention of lymphedema and early intervention for lymphedema is a future challenge, for which a scale to determine the impact of lymphedema is needed [1, 19].

The Functional Assessment of Cancer Therapy (FACT) questionnaire is often used to assess QOL as a measure of the overall health of cancer patients [20]. Other current methods for assessing patient QOL include the SF-36, DASH, and the EORTC QOL-C30. These are self-report questionnaire tools for determining arm and shoulder disability and the impact of lymphedema on physical, functional, and social aspects. However, there has been concern that these assessment tools may not adequately assess breast cancer-related lymphedema, upper limb dysfunction, and sensory impairment. [21–24]. Concerns that this did not accurately reflect the QOL of cancer patients led to the creation of the FACT-B, which focused more on breast cancer treatment, and the FACT-B + 4, which included four additional questions on arm mobility [25–27]. FACT-B + 4 has been translated and used in 27 countries around the world for the evaluation of postoperative lymphedema in breast cancer. However, there is no Japanese translation of FACT-B + 4.

Therefore, we initiated the translation of the FACT-B + 4 in accordance with established translation processes, and report that we have verified the linguistic validity and created a Japanese version of the FACT-B + 4 which has been approved by FACT.

## Methods

### Overview

The translation process was prepared and approved in cooperation with the “FACIT Trans Team (FACIT)” in accordance with international guidelines for translation procedures. The translation process consisted of three steps: (1) forward translations, back translations, and preparation of a provisional Japanese version; (2) pre-testing with the help of five breast cancer patients; and (3) revisions based on pre-test results and clinical perspectives, and final approval. The Japanese translation was created after linguistic validation of the Japanese version (Table 1).

**Table 1:** The translation process follows the international translation guidelines provided by the FACIT Trans Team

1. Forward translation; Translate from English into Japanese and this translation should be provided by a native Japanese
2. Reconciler; Once you have Fwd1 and Fwd2 translations, you’ll need to send it to a third translator. He is also a native Japanese speaker, who will review the English source and the two forward translations and either choose one of the translations or choose a new translation, depending on what that person thinks is most appropriate
3. Back translation; Send the reconciler version only to a fourth translator (do not send the English original), who will provide a back translation (BT) of the item from Japanese back into English. The back-translator should be a native English speaker, if possible
4. FACIT Comments; They will review the steps, as well as the back translations, and provide FACIT comments
5. Review FACIT Comments and Finalized Translation
6. Literal Back translation of final; This is meant to be word-for-word translation: the English translation of the Final that upholds the exact or primary meaning of each word used in the item
7. Polished Back translation of final; This is meant to be the English translation of the Final that reflects what the item is saying in essence. This is what patients who speak the target language will understand when they read this item
8. Harmony check, Debrief notes and Post Test Final

The implementation period was from September 2022 to June 2023, and all authors of this paper were involved to the same extent in the review of the forward translation and the preparation of the Japanese draft, the review of the back-translation and the preparation of the tentative Japanese translation, the review of the pre-test results, and the proposal of the final Japanese translation.

### Forward translation, back-translation, revision, and preparation of a tentative Japanese version

After the creation of the Japanese draft through forward translation, back-translation, and discussions between the developers of the original version and our research team, the Japanese draft was approved by FACT in June 2023.

First, two native Japanese speakers independently prepared Japanese translations of FACT-B + 4. A third translator created a temporary forward translation based on these Japanese translations. FACIT required that “The back-translator should be a native English speaker”. Therefore, we asked two people who were based in the U.S. and familiar with the English language to be the back-translators. For the back-translation, the original English text was withheld, and only the Japanese translation determined by forward translation was disclosed to them, and they back-translated the Japanese text into English.

The results were returned to FACT and evaluated by the secretariat as to whether the back-translated English deviated from the original version or not. Based on the results, the Japanese version was reviewed and revised by the co-authors, and a Japanese translation was prepared in response to FACT’s review. Then, the Japanese translation was prepared in response to the FACT review, and a literal back-translation (“a word-for-word translation: the English translation of the Final that upholds the exact or primary meaning of each word used in the item”) and a polished back-translation (“an English translation of the Final that reflects what the item is saying in essence. This is what patients who speak the target language will understand when they read this item”). The Japanese version was completed, and this process was repeated for all revisions of the Japanese draft, and a provisional Japanese translation was prepared.

### Harmony check, debrief notes, and post-test final version

Cognitive debriefing was conducted as a pre-test for potential users of the tentative Japanese version. Cognitive debriefing is an interview process to confirm that the meaning of the questions is understood and culturally acceptable, and was conducted by the principal investigator through individual interviews with five breast cancer patients who volunteered to participate in the study. Specifically, we asked the patients to rephrase the questions and choices in the tentative Japanese translation in their own words to confirm their understanding of the text and language, and to provide feedback on the difficulty of answering the questions and the difficulty of understanding the text.

Based on the results of the pre-test, the Japanese translation was subjected to a final check by FACT to ensure that it did not deviate from the purpose and meaning of the original version of FACT-B + 4, and received final approval for use in clinical studies.

**Results.**

**Fig. 1:**
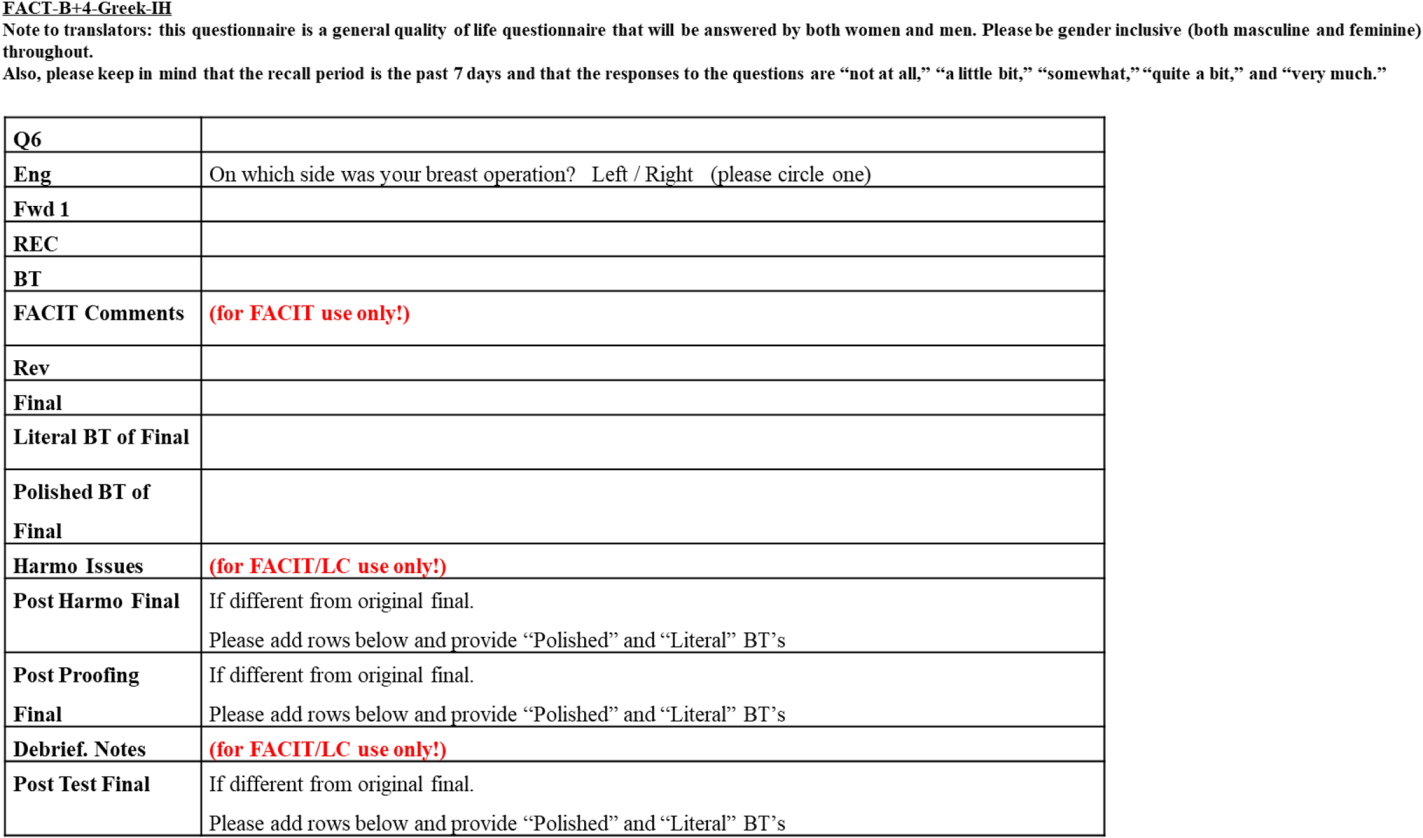
We perform translation using this word spreadsheet

**Fig. 2:**
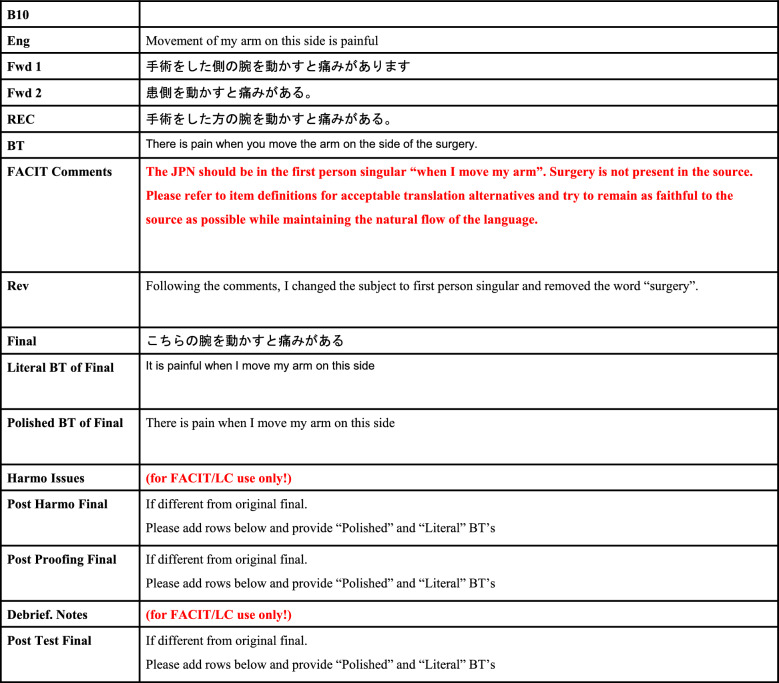
This is the actual translation process of “Movement of my arm on this side is painful"

We worked with FACIT to translate the text, using spreadsheets of words created based on international translation guidelines (Fig. 1). The steps of the translation process and the results are shown in Fig. 2. In this section, the process of translation is illustrated using the phrase “Movement of my arm on this side is painful” from FACT-B + 4 as an example.

### Forward translations

Two native Japanese speakers prepared Japanese translations of the questionnaire items. A third translator created a temporary forward translation based on these Japanese translations. As a result, it was decided that “I feel pain when I move the arm of the person who had the surgery” was appropriate.

### Back translations

Since “The back-translator should be a native English speaker, if possible” was required for this course, we asked two persons who currently live and work in the United States for more than 7 years (with native-level English, of course) to perform the back-translation. The original English text was withheld, and only the Japanese translation determined by forward translation was made available to them, and they back-translated it into English. As a result, a back-translation of “There is pain when you move the arm on the side of the surgery”.

### The results were returned to FACT and evaluated by the secretariat

All translations were requested to faithfully translate the original words, verbs, and nouns. In addition, difficulty with Japanese translations, such as “numb” and “stiffness”, and difficulty in paraphrasing the question sentences were reported. Based on these results, the co-authors reviewed and revised the Japanese version again, and the revised Japanese version was evaluated by FACIT with feedback. After that, a literal back-translation and a polished back-translation were prepared, and the revised translation was evaluated as a tentative translation without problem.

### Harmony check

Considering the process that led to the creation of the provisional Japanese version of FACT-B + 4, FACIT requested a harmonization check to pilot test it with five breast cancer patients. To carry out the harmonization check, a pilot test was conducted with five breast cancer patients using the provisional Japanese version of FACT-B + 4. Because of they are recruited as volunteers, they were met without compensation. Afterward, they were asked to complete a questionnaire to verify its linguistic validity. We recruited volunteers from Okayama University Hospital who had undergone breast cancer surgery and were willing to assist in the creation of the Japanese translation. A pre-test was conducted with the patients who agreed to participate in this project.

The characteristics of these patients are detailed in Table 2 and include the following: age, breast cancer surgery method, history of breast cancer drug therapy treatment, and years since breast cancer surgery.

**Table 2:** Characteristic of patients who participated in this pilot test

	Age	Breast cancer side	Clinical stage	Subtype	Neoadjuvant chemotherapy	Breast surgery	Adjuvant therapy
1	49	Left	T1bN0M0	Triple negative	No	Bp + SNB	RT
2	43	Left	TisN0M0	Luminal	No	Bp + SNB	RT
3	49	Left	T2N0M0	HER2 positive	Yes	Bt + SNB	Trastuzumab
4	72	Left	T1cN0M0	Luminal	No	Bp + SNB	AI + RT
5	59	Right	T1cN1M0	Luminal	Yes	Bt + Ax	AI + CDK4/6

**Fig. 3:**
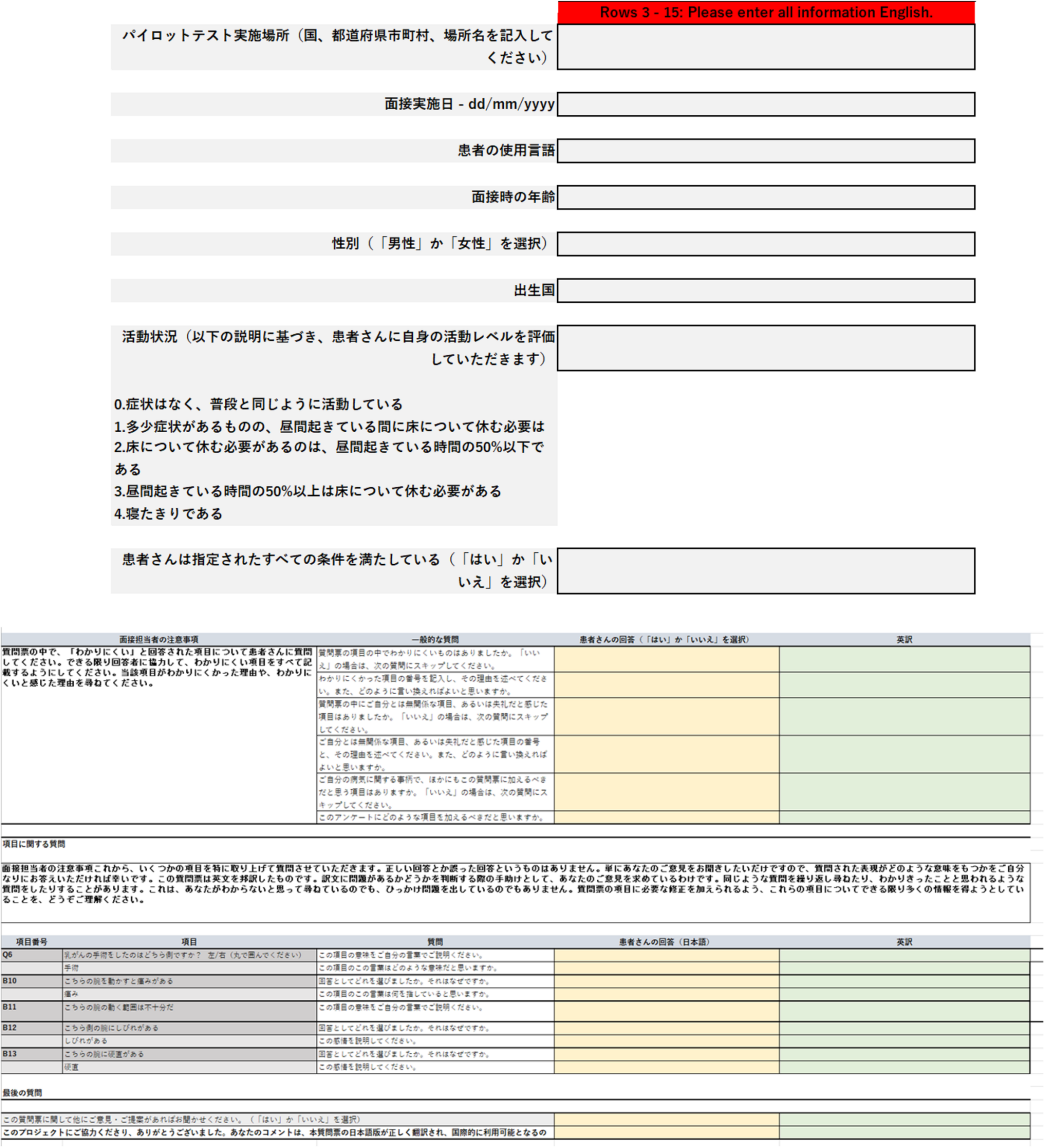
The contents of the harmony check question

The contents of the questionnaire are shown in Fig. 3. The questions used in the harmony check were as follows, and included questions about the clarity of the Japanese text in the provisional Japanese version of FACT-B + 4, as well as questions about the respondents’ interpretations of the Japanese and the reasons for their answers in the questionnaire (Fig. 3).

Questions about the clarity of the Japanese text in the provisional Japanese version of FACT-B + 4.

・Did you find any of the items in the questionnaire difficult to understand?

・Did you find any items in the questionnaire that were not relevant to you or that you felt were rude?

Questions about the respondents’ interpretations of the Japanese and the reasons for their answers.

・Q6: What do you think is the meaning of this item in the Japanese version of FACT-B + 4?

・Q6: Please explain in your own words the meaning of this item, what do you think this word surgery means?

・B10: Which of the following did you choose as your answer? Why is that? Pain: What do you think this word means?

・B11: Please explain the meaning of this item in your own words.

・B12: I have numbness in my arm on this side. Which of the following did you choose as your answer and why?

・B13: I have numbness on this side of my arm.

・B13: There is stiffness in this arm. Which of the following did you choose as your response? And explain this feeling.

**Fig. 4:**
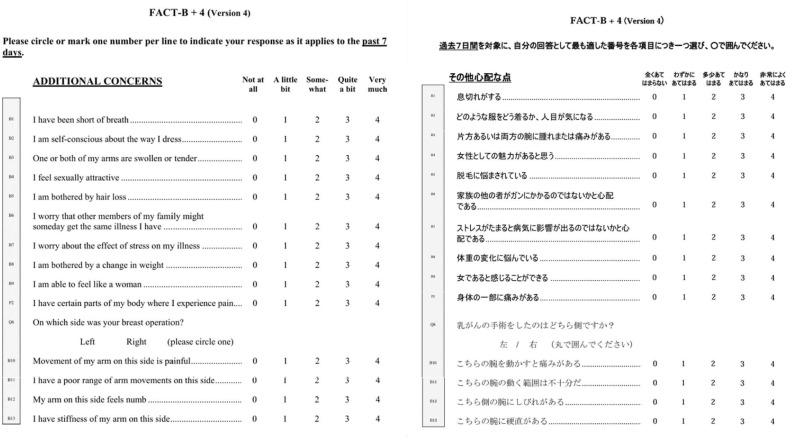
Comparison of the Japanese version of FACT-B + 4 approved by FACIT and the original version of FACT-B + 4

As a result, the patients reported difficulty with Japanese translations, such as “numb” and “stiffness”, and difficulty in paraphrasing the questions. Finally, based on the preliminary test results, the original developer, the Japanese translation team, and the back-translation team reviewed the proposed revisions, made minor modifications to make the wording more concise and closer to everyday language, and created the final Japanese translation, which was approved by FACIT (Fig. 4). As a result, the Japanese version was approved for use in the FACT-B + 4 website, and the Japanese version was serialized in the Language Availability section of the FACT-B + 4 website.

## Discussion

There are several tools commonly used to assess upper limb function and quality of life (QOL), including Quick DASH, FACT-B + 4, EORTC QLQ-C30, and SF-36. Quick DASH and SF-36 are designed to assess upper limb impairment and disability in patients with upper limb problems or disorders affecting the upper limb, with a particular focus on upper limb-related pain, range of motion, and functional ability in daily living. In contrast, EORTC QLQ-C30 and FACT-B + 4 are comprehensive tools used to assess the QOL of breast cancer patients, encompassing multiple questions that address physical, social, emotional, and functional aspects. FACT-B + 4 is an enhanced version of FACT-B, specifically modified to more accurately assess side effects related to breast cancer surgery, such as upper limb swelling and pain.

In accordance with international guidelines for translation procedures, we created the Japanese translation together with the original developer team (FACIT) and obtained approval for use of the Japanese version of FACT-B + 4. On the FACT-B + 4 website, they mention that “Available translations of the FACT-B + 4 can be obtained by registering for permission. Users are not permitted to translate the FACT-B + 4 without permission from FACIT.org. Translations must undergo a rigorous methodology under the guidance of FACIT.org which includes multiple translators, quality assurance steps and cognitive interviews with patients.” Including the Japanese version, translations have already been created in 29 languages (FACT-B + 4 (facit.org)).

In creating the Japanese translation version, there were many instances where it was pointed out that the translation we considered appropriate did not directly translate the original language of the FACT-B + 4, and it was considered most important that the words of the tool be translated directly into Japanese, so that there would be no differences among the tools rather than just making the translation easy to understand. We found that receiving direct feedback from the potential target population of the scale through pre-testing was extremely important to ensure the linguistic validity of the translation. In other words, it was [21] and polished translation, which is carefully adapted to the cultural background and everyday terminology of the country in question.

Japanese have compulsory education up to junior high school, and it is considered easy for them to understand the Japanese translation of the FACT-B + 4 and answer the survey items. Therefore, when we performed the pre-test on five breast cancer patients to determine whether there were any items on the questionnaire that were difficult to understand, all answered that there were no items that were difficult to understand. However, on the question “What do you think this word means” to verify linguistic validity, all participants seemed to give similar answers to the meanings of everyday words, such as “surgery” and “pain,” but for words that are not often used in everyday life, such as “numbness” and “stiffness,” they gave abstract answers. This included onomatopoeia, such as “tingly” and “prickly” for “numbness”, and for “stiffness”, they gave answers that included subjective opinions, such as “uncomfortable, painful, difficult to move”.

We need to pay attention that this FACT-B + 4 Japanese version has been verified for linguistic validity. In other words, although this FACT-B + 4 Japanese version is evaluated as adequately reflecting the original text linguistically, it may not yet be fully proven whether the scale obtained from this Japanese version shows the same scale as the FACT-B + 4 in other countries. In the future, it will be necessary to conduct validation studies and examine the consistency with the original English version. In other words, to fully ensure that there is cross-cultural validity of the Japanese version of the scale developed in this study, it will be necessary to secure a certain sample size and conduct statistical validation, including an examination of the distribution of SCRQoL and its related factors. In addition, the weighting of the questions may be unique to the Japanese version, which is a topic for future research.

## Conclusion

In accordance with international guidelines for translation procedures, a Japanese translation of FACT-B + 4 was prepared, which was approved by the original developer. The importance of obtaining direct feedback from users was confirmed in ensuring the linguistic validity of the translation. We believe that it is important to conduct a validation study of the Japanese translation by conducting a prospective study in the future.
